# Outcomes of implant-based oral rehabilitation in head and neck oncology patients—a retrospective evaluation of a large, single regional service cohort

**DOI:** 10.1186/s40729-019-0161-y

**Published:** 2019-03-05

**Authors:** Dominic P. Laverty, Owen Addison, Berhanu A. Wubie, Giseon Heo, Sat Parmar, Timothy Martin, Prav Praveen, David Pearson, David Newsum, Michael Murphy, Geoffrey Bateman

**Affiliations:** 1 University of Birmingham School of Dentistry, Birmingham Dental Hospital, 5 Mill Pool Way, Birmingham, B5 7EG UK; 2 Birmingham Community Healthcare NHS Foundation Trust, Birmingham, B5 7EG UK; 3 School of Dentistry, University of Alberta, Edmonton, AB T6G 1C9 Canada; 4 Department of Mathematical & Statistical Sciences, University of Alberta, Edmonton, AB T6G 1C9 Canada; 5 University Hospitals Birmingham NHS Foundation Trust, Birmingham, UK; 6 University Hospitals Coventry and Warwickshire NHS Trust, Coventry, UK

**Keywords:** Dental implant survival, Head and neck oncology, Autogenous bone graft, Microvascular free flap, Prosthodontics

## Abstract

**Background:**

The study reports on implant survival outcomes in head and neck cancer patients who received implant-based oral rehabilitation in a regional service centre.

**Methods:**

A retrospective analysis of implant survival outcomes in patients treated in a regional service from 2012 to 2017 was performed. The primary outcome measure was implant survival. The secondary outcome measure was to assess the effect of covariates associated with implant failure including bone type, radiotherapy, chemotherapy, gender and surgical implant complications. Kaplan-Meier survival curves were applied to compare differences in the survival rates of groups of variables. Cox proportional hazards models were applied to identify covariates associated with implant failure. *p* value was set at 0.05.

**Results:**

The sample was composed of 167 head and neck cancer patients who had 779 dental implants placed. Implant survival estimates were calculated: 3 years, 95.7% [95%CI 94.3–97.2%] and 5 years, 95.5% [95%CI 93.9–97.0%], with a median follow-up of 38 months. Gender (*p* = 0.09), radiotherapy (*p* = 0.16) and chemotherapy (*p* = 0.17) did not significantly influence implant survival, whereas implant failure was higher in transported (reconstructed) bone sites in comparison with native bone (*p* < 0.01).

**Conclusion:**

The result of this study suggests that overall implant survival as part of the routine oral rehabilitation is high in this patient cohort; however, implant failure was found to be statistically higher for implant placed into transported bone in comparison to native bone.

## Background

Oral rehabilitation with implant-retained prostheses can significantly improve the quality of life (QoL) for patients following the surgical management of head and neck (H&N) cancer [1], and this treatment modality is becoming more commonly used in this patient group [2–4]. Patients with H&N cancers often undergo ablative surgery with or without reconstruction, radiotherapy and chemotherapy [5]. Such surgical interventions can lead to significant disability, including facial deformity, loss of oral hard and soft tissues, impaired speech, swallowing and mastication [6, 7]. Neither reconstructive surgeries nor conventional prosthodontic techniques are capable of addressing all of these problems successfully [7–9].

Oral and dental rehabilitation is provided to help facilitate mastication, facial support, oral comfort and oral competence and allow patients to speak, chew and appear in public with confidence [6, 10]. Rehabilitation with a removable prosthesis can often be difficult, if not impossible in some patients following surgical management of their H&N oncology. This is due to altered post-surgical anatomy, low salivary flow and a lack of emotional resilience of the patient [6]. For many years, removable prostheses have been central to conventional prosthodontic treatments; however, they have limited success and fail to address all of the problems that the patient may be facing [2, 5, 6, 10–12]. In many cases, a prosthesis may be provided for an aesthetic improvement only, with accepted limited function [6]. The use of osseointegrated dental implants has allowed improved retention of removable prostheses, reduced loading on vulnerable tissues and with this resulted in a reported improvement in the QoL for patients [2, 7, 13, 14].

Osseointegrated dental implants as a treatment modality have been shown to have high success and survival [15]. However, the reliability, safety and usefulness of implant placement in the H&N cancer population remains incompletely defined, mainly due to the limited availability of large, well-constructed studies in the literature [16]. The vast majority of evidence available, to guide clinicians, is formed from case reports and case series, using low patient numbers. Furthermore, the data is universally retrospective in nature which can be understood, as the service provided to this patient group does not lend itself to well-designed highly controlled trials.

With the increasing use of dental implants in the oral rehabilitation of H&N cancer patients [17], an improved evidence base is required to help inform clinical decision-making. The primary objective of this study is to present implant survival rates as part of a service evaluation of large H&N cancer patient cohort, where a consistent care pathway for oral and dental rehabilitation has been operative for the past 5 years. The cohort includes patients whose osseointegrated implants have been placed into a variety of bone types including native, native resected, autogenous non-vascularised and autogenous vascularised bone/free flaps. The secondary objectives are to assess the effect of covariates associated with implant failure such as radiotherapy and chemotherapy, which are frequently eluded to as prognostic factors for implant survival, and also to report the surgical complications during implant placement documented in this patient group.

## Methods

### Study design and setting

The service evaluation was performed by retrospectively examining treatment records of H&N oncology patients who were provided with an implant-retained prosthesis as part of an oral and dental rehabilitation. The study sample was taken from a population of H&N oncology patients that attended the Restorative Dentistry department at Birmingham Dental Hospital (BDH), Birmingham, UK (United Kingdom), for care following primary management of their H&N cancer, in a 55-month period from November 2012 to May 2017. The H&N restorative service provided at BDH is a tertiary care service which covers a population of 5.5 million people within the West Midlands region of the UK. The service was led by a single specialist clinical lead during this period, and treatment was provided at no cost to the patients. Treatments were linked with Oral and Maxillofacial surgical (OMFS) teams at BDH or at University Hospitals Birmingham (UHB), Birmingham, UK. Despite the variability in disease presentation and in its management, a consistent co-ordinated care pathway leading to oral and dental rehabilitation including multi-disciplinary team (MDT) planning was followed. The treatment period for data collection included the care of patients who had received implant-based reconstructions within the same service at an earlier date but required prosthodontic maintenance or revision. These patients were included in the analysis subject to the completeness of the minimum data set.

All H&N oncology patients who had completed an oral rehabilitation that included the use of dental implants to retain a prosthesis, during the census period, were included. Patients were excluded if the minimum data set could not be collected. Restoration of the dental implant with a definitive prosthesis was the criterion for successful completion of the oral rehabilitation in this study.

Approval for this service evaluation was given by the Birmingham Community Healthcare NHS Foundation Trust R&D team (Birmingham, UK).

### Eligibility criteria

#### Inclusion criteria

1. Patients who had suffered with H&N cancer
2. Patients who completed an oral rehabilitation with an implant-retained intra-oral prosthesis
3. Patients who had been followed up on at least one occasion after placement of dental implants

#### Exclusion criteria

1. Patients who did not suffer with H&N cancer
2. Patients who did not complete an oral rehabilitation with an implant-retained intra-oral prosthesis
3. Patients who were not followed up after dental implant placement
4. Patients in whom the minimum data set could not be collected.

### Study variables

The minimum data set required for study inclusion required patient demographics (age, gender); tumour diagnosis; the oncological treatment carried out in the form of surgery (tumour ablation, reconstruction), radiotherapy (field and timing) and/or chemotherapy (drugs); adjunctive surgeries (implant site augmentation); location of implant placement (maxilla, mandible, native bone, resected native bone, autogenous bone grafts vascularised and non-vascularised); dental rehabilitation (fixed, removable and timing) and the implant system used.

The primary outcome of this retrospective study was to assess the survival of dental implants in this patient group (at the patient level), and the secondary objective was to identify possible covariates on implant failure.

### Data collection

Patients were identified from electronic patient management systems (iSoft Patient Manager (iPM) software, RiO (Servelec HSC)). The case notes of all potential patients were retrieved and reviewed at BDH. Records were comprised of a combination of paper medical records, scanned paper medical records (Iron Mountain Digital Record centre) and electronic medical records (Case Stream R4 Clinical+ Practice Management Software). In addition, the clinical notes of all patients were also reviewed at the UHB where primary management of their H&N cancer was undertaken using an electronic patient record system (Clinical Portal). Data were collected from the point of implant planning up until their most recent review appointment either at BDH or UHB.

Data were extracted in an anonymised format to a Microsoft Excel template. Data included gender, age, oncological diagnosis and TNM classification and staging; whether the patient had surgery; radiotherapy (dose and site); chemotherapy (drug types and dosages), nature of the surgical reconstruction and type of microvascular free flap/graft used; types of imagery taken for implant planning; whether surgical guides were used at the time of implant placement; the number of implants used; the sites of the implants placed; the types of bone into which the implants were placed; any documented surgical complications; the team who placed the implant(s); the date(s) of implant placement; the date(s) of implant failure; the number of implant failures and the clinically defined reasons for implant failure; the implant manufacturer and fixture dimensions; the site of the oral rehabilitation and whether the oral rehabilitation was fixed or removable. Finally, the date of the last follow-up was recorded or where appropriate the date of death.

For the purpose of this service evaluation, implant survival was defined as an implant fixture still in situ and implant failure defined as implant fixture not in situ which had been lost or removed for whatever reason. Implant survival time was defined as the time interval from the date of implant placement to the date of implant failure or the last follow-up date, whichever occurred first.

### Implant planning

The majority of patients were planned for implant-based rehabilitation by a specialist restorative dentist in consultation with surgical teams from BDH and UHB. In the Birmingham service, patients are only provided with implants when conventional non-implant-retained prostheses are deemed inappropriate. As part of consent, patients understood the amount of time it would take for the planning, placement and restoring of dental implants and the need for multi-stage treatment and for regular review. All treatment costs were met by the service provider. Radiographic images were taken to assist in planning and included cone beam computed tomography (CBCT) with or without reformatting for implant planning software (SIMPLANT® Computer-Guided Implant Treatment Software (Dentsply Sirona, York, PN, USA) and conventional radiographs.

### Surgical implant placement technique

Implants were placed by experienced surgical and restorative dental teams accustomed to placing a variety of implant systems in this patient group. Implants were placed into the native mandible/maxilla, resected mandible/maxilla or autogenous bone grafts. Implants were placed either free hand or using a surgical implant guide. Implant placement was both primary (at the time of surgical resection/reconstruction) or secondary/delayed (after surgical resection/reconstruction); however, within this service, primary implant placement was uncommon. At the time of restoring or uncovering the implants, the stability of the implants was assessed (manually). Any unstable implants were removed, not used or buried to allow a longer healing time and then potentially used at a later date. Any soft tissue modifications such as further free flap skin paddle debulking and sulcoplasty to provide a sulcus were carried out prior to oral prosthodontic reconstruction, usually at the time of implant placement.

### Statistical approach

Statistical analyses using Kaplan-Meier survival curves were applied to compare differences in the survival rates of groups of variables. The log-rank test method was used to evaluate for significance of differences between groups of covariates on time to failure of implants. A Cox proportional hazards model was applied to identify the covariates associated with the time to failure of implants. The statistical analysis (*ɑ* = 0.05) was conducted considering the patients as the unit of analysis for patient-based variables (gender, chemotherapy, radiotherapy) and with the implant as the unit of analysis for nature of the implant site. Patients that died during the observational period were included in the analysis, but their data was censored beyond the date of their last follow-up appointment. Data were analysed using the statistical analysis software R version 3.3.2.

## Results

### Demographics

**Fig. 1:**
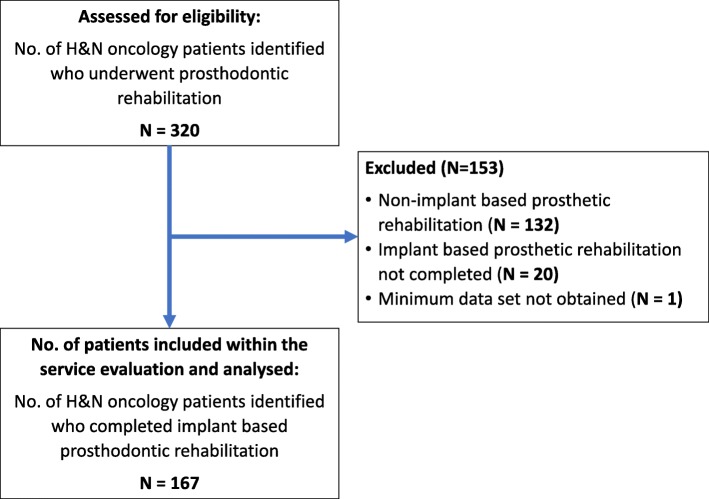
Flow diagram

A total of 167 patients who had undergone implant-based oral rehabilitation from November 2012 to May 2017 were included in this service evaluation (Fig. 1). The study population comprised of 58 women (35%) and 109 men (65%) with a mean age of 63.2 years (range 27–88 years). The 167 patients had a variety of malignant and benign H&N tumours at various sites and stagings (Tables 1 and 2). Patients (from date of implant placement to their most recent review) were followed up for a median of 38 months (range 1–142 months). Seven hundred seventy-nine implants in total were placed in 167 patients. One hundred twenty-four patients had 583 implants placed at UHB, and 43 patients had 196 implants placed by at the BDH. A total of 148 patients (89%) had resective surgery, and of these, 92 patients had reconstructive surgery (55%) with a variety of microvascular free flaps and autogenous bone grafts as shown in Table 3 (note that a single patient received both an anterolateral thigh flap (ALT) and a fibula free flap (FFF) reconstruction). During the observation period, 28 patients included within this service evaluation died. As such, their data was censored from any further analysis beyond the date of their last follow-up appointment.

**Table 1:** Summary of cancer type and site of the study population

Cancer type	No. of patients										
	Buccal	FOM	Mandible	Maxilla	Nasal	Tonsil	Skin	Tongue	Pharynx	Not specified	Total
SCC	8	14	23	24	3	19	2	27	8	0	128
Adenoid cystic carcinoma	0	1	1	5	0	0	0	0	0	0	7
Ameloblastoma	0	0	5	2	0	0	0	0	0	0	7
Unspecified carcinoma/tumour	0	0	1	2	0	0	0	0	1	1	5
Malignant melanoma	0	0	1	1	0	0	0	0	0	1	3
Osteogenic sarcoma	0	0	1	2	0	0	0	0	0	0	3
Mucoepidermoid	0	0	1	1	0	0	0	0	0	0	2
Pleomorphic adenoma	0	0	0	2	0	0	0	0	0	0	2
BCC	0	0	0	0	1	0	1	0	0	0	2
Adenocarcinoma	0	0	0	2	0	0	0	0	0	0	2
Primitive neuroectodermal Tumour	0	0	1	0	0	0	0	0	0	0	1
Chondrosarcoma	0	0	0	1	0	0	0	0	0	0	1
Odontogenic keratinocyst	0	0	1	0	0	0	0	0	0	0	1
Lymphoma	0	0	0	0	0	0	0	1	0	0	1
Dendritic cell sarcoma	0	0	0	1	0	0	0	0	0	0	1
Pindburg tumour	0	0	1	0	0	0	0	0	0	0	1
Total	8	15	36	43	4	19	3	28	9	2	167

A summary of the head and neck cancer diagnoses and anatomical sites within the study population. *FOM* floor of the mouth, *BCC* basal cell carcinoma, *SCC* squamous cell carcinoma

**Table 2:** Description of cancer staging and implant failures

Cancer staging	No. of patients	No. of patients with implant failure	Patient implant failure (%)
I	22	1	4.5
II	20	3	15.0
III	12	2	16.7
IVA	63	12	19.0
IVB	1	0	0
IVC	1	0	0
Unknown	48	6	12.5
Total	167	24	14.4

Description of cancer staging and implant failures at the patient level

**Table 3:** Summary of surgical interventions and tissue type used for head and neck reconstruction

Surgical intervention	No. of patients
No surgery	19
Surgery and no reconstruction	56
Surgery and reconstruction with free flap/autogenous bone graft	92
Total	167
Reconstructive tissue used	No. of patients
Fibula	31
Radial	30
DCIA	11
Scapula	9
ALT	7
Iliac crest (non-vascular)	3
Pectoralis Major	2
Total	93

Cancer staging and the number and percentages of patients experiencing implant failure for each cancer stage (where applicable). TNM tumour, node, metastasis

### Implant imaging and planning

One hundred thirty-eight patients (83%) had a CBCT scan taken and reformatted for SIMPLANT® for implant planning purposes; once planned, this scan was used to construct SIMPLANT® Surgical Guides (Dentsply Sirona, York, PN, USA) for use at the time of surgical implant placement. For two patients, CBCTs were taken for implant planning (in both these cases, these acquired CBCTs were not reformatted for use with SIMPLANT® planning software); 23 patients had conventional plain radiographs taken for planning, and for four patients, it was unclear what radiographic imagery were taken for implant planning purposes.

### Implants

A variety of implant systems were used which included 679 Straumann (Institut Straumann, Basel, Switzerland) implants, 63 Brånemark (Nobel Biocare, Zurich, Switzerland) implants, 36 Astra Tech (Dentsply Implants, Mannheim, Germany) Implants and one Oktagon (Dental Ratio, Langenfeld, Germany) implant, with a range of one to 11 implant used per patient. Of these, 373 (48%) implants were placed in the maxilla and 406 (52%) implants in the mandible (Table 4). Ten patients had primary implant placement with 26 implants, and 157 patients had secondary/delayed placement with 753 implants. Implants were placed into either non-resective native bone, resected native bone (which has not been reconstructed) or free flaps/autogenous bone grafts. Of the 92 patients who received reconstructive surgery with microvascular free flaps/autogenous grafted bone, 52 patients had implants placed into these reconstructed sites with 129 implants placed. In the remaining patients, 22 implants were placed into resected native bone (which has not been reconstructed) and 628 implants placed into non-resected native bone with 323 implants in non-resected native mandible and 305 in non-resected native maxilla.

**Table 4:** Implant survival in specified bone type

Bone type	No. of implants	No. of implant failures	Implant survival (%)
All patients	779	34	95.6
Native maxilla/mandible (non-resected)	628	12	98.0
Native mandible (non-resected)	323	7	97.8
Native maxilla (non-resected)	305	5	98.4
Resected mandible/maxilla not grafted with autogenous bone	22	0	100
Native autogenous bone graft	129	22	82.9

Implant numbers, failures and implant survival percentages overall and divided into each type of bone into which the implants were placed which include; native bone, resected native bone and autogenous bone graft sites

### Radiotherapy and chemotherapy

A total of 105 patients (63%) received some form of radiotherapy with or without chemotherapy. Of these, 75 patients received radiotherapy (45%), 30 patients received chemoradiotherapy (18%) and no patients received chemotherapy in isolation (Table 5). Due to the retrospective nature of the study, the precise radiation fields could not be obtained in 30 patients and, therefore, it was not possible to estimate dosimetry to each of the implant sites. In the 75 patients in whom radiation fields were documented, the radiation dose for therapeutic radiotherapy ranged from 50 to 70 Gy in 72 patients. Two patients received palliative radiotherapy at 30 Gy with one of these patients stopping at a 7.5-Gy dose due to radiation-related complications and one patient received a higher dose of 88 Gy. A variety of adjunct chemotherapy drugs were used in 30 patients and shown in Table 6.

**Table 5:** Use and timing of radiotherapy, chemotherapy and implant failure

	No. of patients	No. of implants	No. of patients with failed implants	Patient-level implant failure (%)	No. of implant failures	Implant level failure (%)
Radiotherapy	75	382	11	14.7	15	3.9
Pre-operative	68	360	8	11.8	9	2.5
Post-operative	7	22	3	42.9	6	27.3
Chemoradiotherapy	30	143	7	23.3	11	7.7
Pre-operative	29	138	7	24.1	11	8.0
Post-operative	1	5	0	0	0	0
Chemotherapy	0	0	0	0	0	0
Neither	62	254	6	9.7	8	3.2
Total	167	779	24	14.4	34	4.4

The number of patients and implants placed into patients who recieved radiotherapy, chemoradiotherapy, chemotherapy (pre- and post-implant placement) and those that or did not receive radiotherpahy or chemotherapy and the number and percentage of patients and implants that failed in each of these groups

**Table 6:** Chemotherapy agents used within the study population

Chemotherapy agents	No. of patients
Carboplatin	13
Cisplatin	10
Cetuximab	2
MAP chemo (methotrexate, doxorubicin, cisplatin)	2
R-CHOP (rituximab, cyclophosphoamide, doxorubicin, vincristine, prednisolone)	1
TPF (docetaxel, cisplatin, 5-fluorouracil)	1
Carboplatin and paclitaxel	1
Total	30

The drugs and regimes of chemotherapy agents used within the study population in the management of their head and neck cancer

### Pre-prosthetic surgery

In total, 19 patients required further surgery prior to oral rehabilitation. Eight patients required debulk of the soft tissue component of the microvascular free flap, ten patients required a sulcoplasty and one patient required surgery to release the tongue and improve its mobility to assist in oral rehabilitation.

### Surgical complications during implant surgery

Surgical complications during the placement of the dental implants were noted in 24 of 167 patients (14.8% of patients). Complications have been categorised as treatment plan related, anatomy related, procedure related and other (according to Misch et al.,) [18] and are summarised in Table 7. Note that when CAD-CAM surgical implant guides (SIMPLANT® Surgical Guides (Dentsply Sirona, York, PN, USA) are referred to, these are from reformatted CBCTs and were planned using SIMPLANT® implant planning software.

**Table 7:** Surgical complications reported during implant placement

Surgical complications	No. of cases
Treatment planning related	
During implant, placement reconstruction screw hit and reconstruction screw were removed to accommodate the implant	2
Implant position was changed during surgical procedure and the implant was placed free hand as the implant position from the surgical guide was deemed inappropriate	2
Anatomy related	
Difficult surgical access to place implants so implants were not placed	2
The implant was not placed as there is a high risk of inferior dental nerve damage	1
CAD-CAM surgical guide made access more challenging so it was not used to prepare posterior sites	1
Lack of bone volume to place implant—so an alternative site was used	3
Large incisions were required to attain surgical access to fit the CAD-CAM surgical guide which was deemed inappropriate and the implants were subsequently placed free hand	1
Procedure related	
Lack of primary stability of the implant so larger implant diameter was used to achieve primary stability	4
Lack of primary stability of the implant—implants left in situ	2
Lack of primary stability of implants—so the implant was not placed	1
Lack of primary stability of the implant—so the implant was placed in an alternative site	1
The implant was not placed due to being placed too deep	1
Other	
Inadequate fit of CAD-CAM surgical guide—either was not used or was used in to estimate the implant bed preparation site and angulation but then prepared and placed free hand	3
CAD-CAM surgical guide needed to be adjusted to allow it to fit	1
Total	24

The number of cases and type of surgical complications that were documented during the process of surgical implant placement in this study population. These were grouped into treatment planning- , anatomy- , procedure-related and other. CAD-CAM computer-aided design-computer-aided manufacture

### Implant failure

**Fig. 2:**
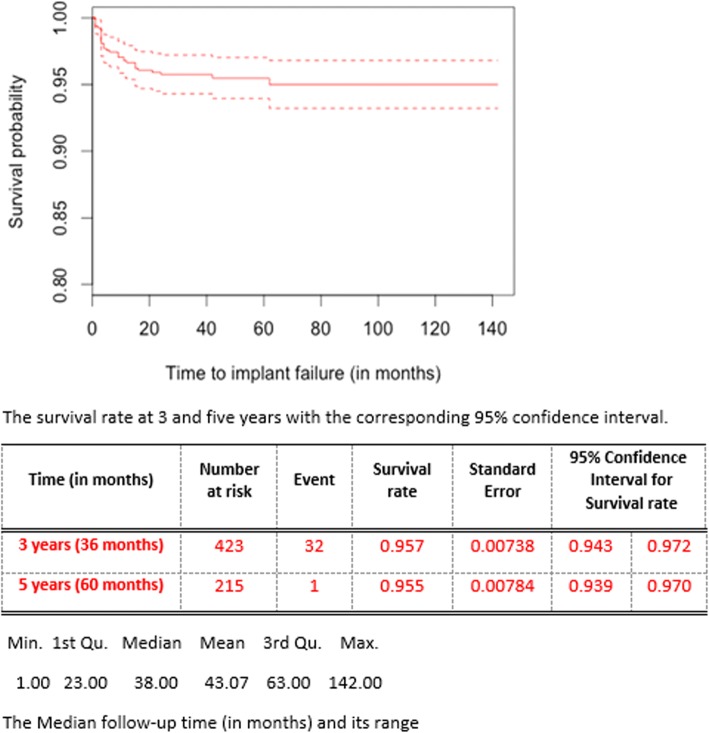
A Kaplan-Meier survival curve for overall implant failure in this patient cohort. Implant survival rate at 3- and 5-year rates with corresponding CIs are shown. Median follow-up time and its range are also shown. CI confidence interval, Min. minimum, 1st Qu. first quartile, 3rd Qu. third quartile, Max. maximum

Thirty-four implant failures were observed out of 779 implants placed (median follow-up of 38 months, mean follow-up of 43 months and a range of 1–142 months). A Kaplan-Meier survival curve for overall implant survival is shown in Fig. 2. The median survival time is not attainable since the survival rate for the overall trend is better than 0.50. Survival rate estimates at 3 years and 5 years were 95.7% [95%CI 94.3–97.2%] and 95.5% [95%CI 93.9–97.0%], respectively.

**Fig. 3:**
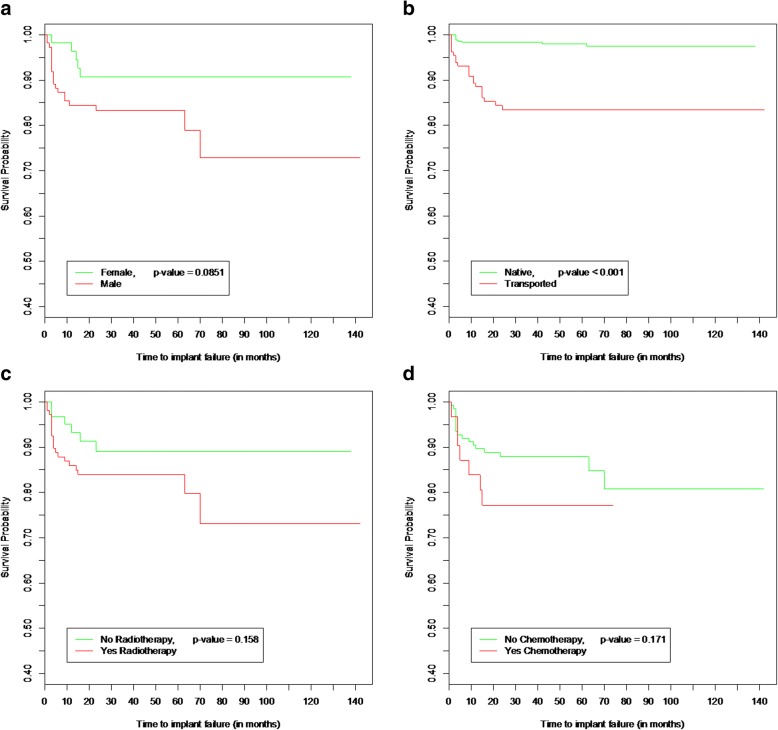
**a** Kaplan-Meier survival curves comparing implant survival according to gender. Implants placed into males had higher failure rate in comparison to those placed into females; however, this was not found to be statistically significant (*p* = 0.09). **b** Kaplan-Meier survival curves comparing implant survival in the native and transported bone. Implants placed into the transported bone had higher implant failure rate in comparison to implants placed into the native bone. This was found to be statistically significant (*p* ≤ 0.001). **c** Kaplan-Meier survival curves comparing implant survival in patients who received radiotherapy with those that did not. Implants placed into patients who received radiotherapy had a higher implant failure rate in comparison to implants placed into those patients who did not; however, this was not found to be statistically significant (*p* = 0.16). **d** Kaplan-Meier survival curves comparing implant survival in patients who received chemotherapy with those that did not. Implants placed into patients who received chemotherapy had a higher implant failure rate in comparison to implants placed into those patients who did not; however, this was not found to be statistically significant (*p* = 0.17)

Implant failure occurred in 24 of the 167 patients included (14.4% failure at a patient level). The mean age of study cohort was 63.2 years, and the mean ages of patients exhibiting implant failure or no failures were similar at 62.7 and 63.3 years, respectively. Of the 58 female patients within this cohort, five experienced implant failure (8.6%) whereas 19 of 109 male patients had implant(s) fail (17.4%) although this was not statistically significant (*p* = 0.09) (Fig. 3a).

### Timing

The 34 implant failures were classified by the stage of treatment in which they failed, where stage II is the surgical uncovering of the implant fixture to allow prosthodontic restoration:

- Prior to stage II—3 implant failures
- At stage II and before prosthetic loading—22 implant failures
- After prosthetic loading—9 implant failures

For the 22 implants (in 17 patients) that failed due to a lack of initial osseointegration, the mean and median time to failure were 140 and 97 days, respectively. The mean and median time to failure of the five implants (in four patients) that failed due to peri-implantitis were 915 and 683 days, respectively. Of the six implants that failed due to free flap failure (in two patients), for one of these patients, failure occurred at day 16 after free flap reconstruction and primary implant placement and the other occurred at 451 days after implant placement when there was late failure (as a result of a pathological fracture due to osteoradionecrosis (ORN)). One implant (in one patient) was explanted as it was deemed to be in an unrestorable position and was causing soft tissue trauma after 366 days.

### Bone type

Implant survival was high for implants placed into native bone (both resected and non-resected) (Table 4). Implant survival for implants placed into autogenous free flaps was 100% in scapula flaps, 83.0% in fibula free flaps (FFF), 80.0% in radial composite free flaps (RFF) and 76.0% in deep circumflex iliac artery flaps (DCIA). Implant survival in non-vascularised iliac bone graft was 80.0%. Implant survival in native bone associated with microvascular soft tissue flaps was 100% for anterolateral thigh flap (ALT). For pectoralis major flaps (PMF), no implant was placed through this soft tissue flap (Table 8). Kaplan-Meier survival curve comparing outcomes of a simplified comparison between implant failure in native and autogenous bone grafts/free flaps is shown in Fig. 3b. A statistically significant difference in implant failure was demonstrated with increased implant loss in transported bone (autogenous bone graft/free flap sites) in comparison to implant loss in native bone (*p* < 0.01). The majority of implant loss events were recorded in the first 6 months in native bone whereas loss in autogenous bone graft site was more progressive up until 24 months.

**Table 8:** Type of microvascular free flap/autogenous bone graft implant placed into and implant survival

Type of microvascular free flap/autogenous bone graft—implant inserted into	No. of patients	No. of implants	No. of implant failures	Implant survival (%)
Scapula	5	12	0	100
Fibula	27	65	11	83.1
ALT	1	2	0	100
Radial	6	15	3	80.0
Pectoralis major	0	0	0	–
DCIA	10	25	6	76.0
Iliac crest (non-vascular)	3	10	2	80
Total	52	129	22	82.9

The number of patients and implants and percentage implant failure and survival for each autogenous bone graft that the implants were placed into. DCIA deep circumflex iliac artery flaps, ALT anterolateral thigh flap

### Radiotherapy and chemotherapy

In total, 105 patients received some form of radiotherapy with 525 implants placed into this patient group. Of these, 18 patients experienced implant failure with 26 implants failing in total with a patient implant failure rate of 17.1% and an implant failure rate of 5.0%. There were 62 patients that received 254 implants that did not receive any radio- or chemoradiotherapy; of these, 6 patients experienced implant failure with 8 implants failing in total with patient implant failure rate of 9.7% and an implant failure rate of 3.2%. Kaplan-Meier survival curves for radiotherapy and chemotherapy are presented in Fig. 3c, d. Both variables were not found using the log-rank test method to statistically have a significant effect on implant survival (*p* = 0.16 radiotherapy, *p* = 0.17 chemotherapy).

For patients receiving a combination of chemotherapy with radiotherapy, a higher implant failure rate than those patients who received radiotherapy without chemotherapy was observed. Thirty patients in total received chemoradiotherapy with 143 implants being placed into this patient group. Eleven implant failures occurred in 7 patients (patient implant failure of 23.3% and an implant failure of 7.7%). This is in comparison with radiotherapy where 75 patients received radiotherapy and 382 implants placed with 15 implant failures occurring in 11 patients (patient implant failure of 14.7% and an implant failure rate of 3.9%) (Table 5). Despite this indication, a fitted Cox PH model for implant failure considering radiotherapy and chemotherapy factors and their combination identified no significant effect. The vast majority of patients received radiotherapy and/or chemotherapy prior to implant placement (Table 5), and therefore, it is not appropriate to discuss timing of these interventions and implant survival within this study.

### Implant system and implant geometry

Implant failure with each implant system was calculated and showed varying failure rates (Table 9); however, it would be inappropriate to draw rigid conclusions from this data due to the small numbers of both patients and implants used with some of the implant systems. The most common implant to fail was Brånemark implants with unknown dimensions with 8 failures; this was followed by Straumann RN 4.1-mm-diameter and 10-mm-length implants with 7 implant failures and Straumann RN 4.1-mm-diameter and 12-mm-length implants with 6 implant failures. However, it would be inappropriate to draw conclusions from this data due to incomplete data (164 implant dimensions/lengths were unknown in the 779 implants placed) and the small numbers of some of the implant dimensions used. No real statistical or descriptive analysis of the implant diameter or length can be drawn, and thus, in this retrospective study, implant length/diameter cannot be considered to affect implant survival.

**Table 9:** Implant system and implant failure

Implant system	No. of patients	No. of implants	No. of implant failures	Implant failure (%)
Straumann	140	679	24	3.5
Brånemark	16	63	8	12.7
Astra Tech	11	36	2	5.6
Oktagon	1	1	0	0.0
TOTAL	168	779	34	96.5

The number of patients, implants placed and implant failures and percentage implant failure for each implant system used in this patient cohort (note: one patient had both Straumann and Brånemark implants placed) (implant manufacturers: Straumann implants (Institut Straumann, Basel, Switzerland), Brånemark implants (Nobel Biocare, Zurich, Switzerland), Astra Tech implants (Dentsply Implants, Mannheim, Germany), Oktagon implants (Dental Ratio, Langenfeld, Germany)

### Cancer staging

Patient-level implant failure for cancer staging was calculated. Data may indicate a correlation between higher cancer staging and increased patient implant failure (Table 2). However, it would be inappropriate to draw rigid conclusions due to the small size of some of the groups.

### Surgical complications

Implant failure was higher when surgical complications were experienced during implant fixture placement. In total, 24 patients experienced surgical complications during implant placement; of these, 9 patients experienced implant failure (37.5% of patients with surgical complications) and led to 12 implant failures in total of the 100 implants that were placed in this patient group (with an implant failure rate of 12% in patients that experienced surgical implant complications). This is higher in comparison with the patients that had no documented surgical complications during implant placement with implant failure occurring in 15 of 143 patients (10.5% of patients with no documented surgical complications) and led to 22 implant failures of the 679 implants that were placed (with an implant failure rate of 3.2% of implants with no documented surgical complications in patients that did not experience surgical implant complications).

## Discussion

The use of dental implants as part of the oral and dental rehabilitation in H&N oncology patients is becoming increasingly popular [19, 20]. Implants enable rehabilitation in patients in whom conventional removable prostheses are not possible or provide an inadequate functional and cosmetic result. A UK national survey of OMFS surgeons’ attitudes in the treatment and dental rehabilitation of oral cancer patients by Alani et al., which compared its finding with a study 15 years previously, reported that the use of dental implants had increased in the use to rehabilitate H&N oncology patients from 43 to 93%, between 1995 and 2009 [17]. The purpose of this article is to present the implant survival rates in a large H&N cancer patient cohort at a regional treatment centre. The results obtained demonstrate that implant survival is high and reliable in this challenging patient group. When comparing the implant survival rate of this study with others, findings appear consistent with previous literature which reports implant survival ranging from 75 to 97.1% with average follow-up ranging from 30.9 months to 5.4 years [5, 10, 12, 16, 21, 22].

In this study, the bone type into which the implants were placed influenced survival. A trend can be observed suggesting higher implant survival when placed within the native mandible/maxilla in comparison with implants placed into autogenous bone grafts and vascularized free flaps. This is consistent with the majority of the reported literature [5, 9, 10, 16]; however, equivalent implant survival in native and autogenous bone grafts/vascularized free flaps has been reported by some centres [23, 24]. Radiotherapy is commonly reported as a risk factor for implant failure. In this study, radiotherapy did not statistically significantly affect implant survival either alone or in combination with chemotherapy. There was, however, a trend towards higher numbers of failures in both of these treatment groups (Fig. 3c, d). In this cohort, the majority of patients received radiotherapy and/or chemotherapy prior to implant placement. The existing evidence base suggests that in particular timing of radiotherapy can effect implant survival, with increased failure reported when radiotherapy is carried out before implant placement [14, 25, 26]. The data quality is however poor, and a systematic review by Nooh concluded that timing of radiation therapy in relation to implant placement had no significant effect on implant survival [27].The combined use of chemoradiotherapy appeared to influence implant survival with a higher implant failure seen in this cohort when compared with patients who received either treatment modality in isolation. This observation supports a report by Hessling et al. [5] who found a statistically significant correlation between implant loss and adjuvant combined radiotherapy and chemotherapy [5].

Patients with higher cancer staging showed a trend towards increased implant failure (at the patient unit of measurement level). However, there is little evidence in the literature to support this with Granström [26] reporting no correlation between tumour type, size, stage, nodes or metastasis and implant outcomes [26]. The complexity of surgery will undoubtedly influence the subsequent environment into which implant placement is planned, and it was clear from this cohort analysis that surgical complications at the time of implant placement were frequent and varied. A trend between implant failure and reports of surgical complications was observed but could not be safely statistically tested due to the large number of covariates and confounding factors. Surprisingly, there appears to be no literature reporting on this concept with which to compare this observation.

Implant survival within this study did not appear to be affected by patient demographics of age or sex. In relation to some of the factors that were considered, definite conclusions could not be reached due to small patient/implant numbers within comparative groups and also the incomplete data capture due to the retrospective nature of this study. This included the implant system and implant dimensions. When assessing the literature with regard to implant dimensions, Buddula et al. [25] and Klein et al [28] reported that implant dimensions had no effect on implant survival [25, 28]; however, these studies had a relatively short follow-up. Shaw et al. [10] on the other hand found that implants of less than 13 mm length had a higher rate of failure over longer implant lengths in this patient group [10].

The major strength of this study is the large patient and implant number with a reasonable follow-up period when compared with the previously literature. Some of the principal limitations of this study are its retrospective nature, the limited follow-up period which unfortunately can be expected in this patient group which is also seen in the literature and also the inability to eliminate confounding variables due to heterogeneity of the patients, treatments and follow-up. When reviewing the literature on implant survival/failure in H&N patients, there is a lack of well-designed prospective studies with long-term follow-up, with the majority of the literature being retrospective with small patient numbers and short follow-up. These studies are hugely variable, and to make an effective comparison is difficult and in some cases inappropriate.

Accordingly, there is a clear need for a standardisation of reporting implant survival and failure. There is reasonable overall agreement on the criteria for implant survival and failure; however, there is no agreed minimum data set for collection to enable the comparison of studies, and furthermore there is no consensus on the best way to measure outcomes, analyse endpoints and the most appropriate way to statistically analyse the data.

## Conclusion

This study reports high implant survival when used as part of the routine oral rehabilitation of H&N oncology patients with a median follow-up of 38 months. Implant survival estimates at 3 years was 95.7% [95%CI 94.3–97.2%] and 95.5% [95%CI 93.9–97.0%] at 5 years. Survival analyses for specific covariates showed trends for increased implant failure in patients receiving radiotherapy (*p* = 0.16), chemotherapy (*p* = 0.17) and being male (*p* = 0.09) but were not found to be statistically significant in this population. Implant survival however was found to be affected by the bone type with implant failure being higher for implants placed into autogenous bone grafts/free flaps in comparison to implants placed into native bone which was found to be statistically significant (*p* ≤ 0.001). Reported surgical complications noted at the time of implant placement were high with 14.8% of patients experiencing such events. Such complications appeared to increase the risk of implant failure (at the patient level).

Overall, this service evaluation supports the use of dental implants in oral rehabilitation of this complex patient group, but it is important to recognise that this is an analysis of a complex care pathway with a large number of confounding variables. The findings should not be considered as generalisable beyond the specific environment in which this study was conducted. However, the findings highlight the urgent need for prospective multi-centre standardised data recording in order to generate robust data to enable potentially important treatment covariates to be explored.

## Abbreviations

1st Qu: First quartile
3rd Qu.: Third quartile
ALT: Anterolateral thigh flap
BCC: Basal cell carcinoma
BDH: Birmingham Dental Hospital
CAD-CAM: Computer-aided design-computer-aided manufacture
CBCT: Cone beam computed tomography
CI: Confidence interval
DCIA: Deep circumflex iliac artery flaps
FFF: Fibula free flap
FOM: Floor of the mouth
H&N: Head and neck
iPM: iSoft Patient Manager
Max.: Maximum
MDT: Multi-disciplinary team
Min.: Minimum
OMFS: Oral and maxillofacial surgical
ORN: Osteoradionecrosis
PMF: Pectoralis major flaps
QoL: Quality of life
RFF: Radial composite free flaps
SCC: Carcinoma; squamous cell carcinoma
TNM: Tumour, node, metastasis
UHB: University Hospitals Birmingham
UK: United Kingdom

## References

[1] Marx RE, Morales MJ (1998). The use of implants in the reconstruction of oral cancer patients. Dent Clin N Am.

[2] Schoen PJ, Reintsema H, Raghoebar GM, Vissink A, Roodenburg JLN (2004). The use of implant retained mandibular prostheses in the oral rehabilitation of head and neck cancer patients. A review and rationale for treatment planning. Oral Oncol.

[3] Müller F, Schädler M, Wahlmann U, Newton JP (2004). The use of implant-supported prostheses in the functional and psychosocial rehabilitation of tumor patients. Int J Prosthodont.

[4] Reintsema H, Oort van RP, Schoen P, Raghoebar GM (1998). Implant reconstructive prostheses in the mandible after ablative surgery: a rationale for treatment planning. J Fac Som Prost.

[5] Hessling SA, Wehrhan F, Schmitt CM, Weber M, Schlittenbauer T, Scheer M (2015). Implant-based rehabilitation in oncology patients can be performed with high long-term success. J Oral Maxillofac Surg.

[6] Barrowman RA, Wilson PR, Wiesenfeld D (2011). Oral rehabilitation with dental implants after cancer treatment. Aust Dent J.

[7] Nelson K, Heberer S, Glatzer C (2007). Survival analysis and clinical evaluation of implant-retained prostheses in oral cancer resection patients over a mean follow-up period of 10 years. J Prosthet Dent.

[8] Sclaroff A, Haughey B, Gay WD, Paniello R (1994). Immediate mandibular reconstruction and placement of dental implants. At the time of ablative surgery. Oral Surg Oral Med Oral Pathol.

[9] Watzinger F, Ewers R, Henninger A, Sudasch G, Babka A, Woelfl G (1996). Endosteal implants in the irradiated lower jaw. J Craniomaxillofac Surg.

[10] Shaw RJ, Sutton AF, Cawood JI (2005). Oral rehabilitation after treatment for head and neck malignancy. Head Neck.

[11] Cuesta-Gil M, Ochandiano Caicoya S, Riba-García F, Duarte Ruiz B, Navarro Cuéllar C, Navarro Vila C (2009). Oral rehabilitation with osseointegrated implants in oncologic patients. J Oral Maxillofac Surg.

[12] Yerit KC, Posch M, Seemann M (2006). Implant survival in mandibles of irradiated oral cancer patients. Clin Oral Implants Res.

[13] Harrison SJ, Stratemann S, Redding WS (2003). Dental implants for patients who have had radiation treatment for head and neck cancer. Spec Care Dentist.

[14] Fierz J, Hallermann W, Mericske-Stern R (2013). Patients with oral tumors. Part 1: prosthetic rehabilitation following tumor resection. Schweiz Monatsschr Zahnmed.

[15] Pjetursson BE, Asgeirsson AG, Zwahlen M, Sailer I (2014). Improvements in implant dentistry over the last decade: comparison of survival and complication rates in older and newer publications. Int J Oral Maxillofac Implants.

[16] Ch'ng S, Skoracki RJ, Selber JC (2016). Osseointegrated implant-based dental rehabilitation in head and neck reconstruction patients. Head Neck.

[17] Alani A, Owens J, Dewan K, Summerwill A (2009). A national survey of oral and maxillofacial surgeons’ attitudes towards the treatment and dental rehabilitation of oral cancer patients. Br Dent J.

[18] Misch K, Wang HL (2008). Implant surgery complications: etiology and treatment. Implant Dent.

[19] Granström G, Tjellström A, Brånemark P, Fornander J (1993). Bone-anchored reconstruction of the irradiated head and neck cancer patient. Otolaryngol Head Neck Surg.

[20] Kildal M, Wei FC, Chang YM, Chen HC, Chang MH (2001). Mandibular reconstruction with fibula osteoseptocutaneous free flap and osseointegrated dental implants. Clin Plast Surg.

[21] Linsen SS, Martini M, Stark H (2012). Long-term results of endosteal implants following radical oral cancer surgery with and without adjuvant radiation therapy. Clin Implant Dent Relat Res.

[22] Teoh KH, Huryn JM, Patel S (2005). Implant prosthodontic rehabilitation of fibula free-flap reconstructed mandibles: a Memorial Sloan-Kettering Cancer Center review of prognostic factors and implant outcomes. Int J Oral Maxillofac Implants.

[23] Schliephake H, Neukam FW, Schmelzeisen R, Wichmann M (1999). Long-term results of endosteal implants used for restoration of oral function after oncologic surgery. Int J Oral Maxillofac Surg.

[24] Chiapasco M, Colletti G, Romeo E, Zaniboni M, Brusati R (2008). Long-term results of mandibular reconstruction with autogenous bone grafts and oral implants after tumor resection. Clin Oral Implants Res.

[25] Buddula A, Assad DA, Salinas TJ, Garces YI, Volz JE, Weaver AL (2012). Survival of dental implants in irradiated head and neck cancer patients: a retrospective analysis. Clin Implant Dent Relat Res.

[26] Granström G (2005). Osseointegration in irradiated cancer patients: an analysis with respect to implant failures. J Oral Maxillofac Surg.

[27] Nooh N (2013). Dental implant survival in irradiated oral cancer patients: a systematic review of the literature. Int J Oral Maxillofac Implants.

[28] Klein MO, Grötz KA, Walter C, Wegener J, Wagner W, Al-Nawas B (2009). Functional rehabilitation of mandibular continuity defects using autologous bone and dental implants - prognostic value of bone origin, radiation therapy and implant dimensions. Eur Surg Res.

